# Treatment of Piles by Injections

**Published:** 1884-10-20

**Authors:** Israel B. Washburn

**Affiliations:** Rensselaer, Indiana


### TREATMENT OF PILES BY INJECTIONS.

By ISRAEL B. WASHBURN, M.D., OF RENSSELAER, INDIANA.

There are few diseases that afflict humanity more painful or more common than piles. They are easily diagnosed, and may be treated with little pain, great success, and the smallest possible per cent. of danger.

Notwithstanding this fact, the disease is illy understood and has been harshly and empirically treated to a degree that has so disgusted those afflicted that it is almost impossible to make them believe that we now have a treatment that is almost painless and yet a certain cure, perhaps not at the first operation but in time.

I am aware that a majority of writers contend that piles are enlargements of the hemorrhoidal veins. Can they not be mistaken? Are they not rather an inflammation of the connective tissue? I suggest this on the authority of one who studied the disease thoroughly.

I started out to give the treatment. The physician will need an anal speculum. The one I use is a bivalve-hinge, sold by John Reynders & Co., and is marked "Collin & Co" It is simple, readily introduced, exposes about one-fourth of the rectal circumference at a single expansion, and is not very uncomfortable to the patient.

There are other instruments that may be used, but I like the one above mentioned.

In using the speculum, the patient, if a lady, may be placed on either side; if a man, on either side or back, the thighs closed, flexed upon the abdomen. If the tumors are very large or tender, it is necessary to inject two or three drachms of olive oil into the rectum to aid the introduction of the instrument. For that purpose, a No. 1 hard rubber female syringe is the best instrument. The instrument should then be oiled with olive oil, or what I consider better is petrolatum or lard. The speculum should be introduced very gently, and as far as possible before dilating it. In dilating the rectum with the hinged instrument it is generally necessary, after dilating the bowel one-half, to allow the instrument to partly close in order that the tumor, if there be one, may drop into the field and show well. If the hemorrhoidal tumors are protruding, they should always be returned, if possible, within the bowel before introducing the speculum and especially before treatment, even if the patient has to take an anæsthetic before they can be put back. Return them first.

If necessary to examine all quarters of the rectum, the speculum must be re-introduced each time the operator desires to examine the anterior, posterior or lateral walls. In withdrawing the instrument it should only be partly closed, or it may catch and pull down a tumor, much to the discomfort of a patient.

Allingham, in his work on Diseases of the Rectum, p. 90, quotes Dr. Matthews, of Louisville, Ky., to prove the operation by the injection of carbolic acid into a pile painful, inefficient, and that death is to be feared from peritonitis, embolism and pyæmia.

I have used, seen used, and known of the use of the acid in scores of cases, with no serious results following.

The principal cause of accidents alluded to by writers is the use of too strong a preparation of the acid.

If I wish to slough out a tumor, I use but one part of the acid to two of sperm oil. If to remove it by absorption, I use one to four.

The operator must not inject a syringefull, but must be governed by whether his patient is easily given pain or not. If he is of nervous temperament, five, ten or fifteen drops will be as much as he can bear, and the operation may have to be repeated two or three times before complete removal of the tumor. In order to prepare the remedy so that the acid and oil will not separate, I dissolve the acid by heat, measure the acid and oil in a minim glass, mixing both in a vial, then place the vial in a vessel containing water and bring the water to about the boiling point, after which they will not separate.

I use a hypodermic syringe with a two-drachm barrel, with a needle about three inches long. In order that the needle be strong, the first two inches should be large with an ordinary hypodermic needle point soldered upon it. The piston should be graduated so the tap on the piston may be fixed at a point when the operator will know just how many drops of the preparation would be used in an operation.

If the patient is very nervous, weak, or easily given pain, six to ten drops of the strongest preparation will be a large dose, while the same amount of the weakest would be all that it would be necessary to use.

When everything is ready for an operation, the syringe loaded, patient in position with tumor exposed, the needle should be inserted so that the fluid would be deposited as near the center of the tumor as possible.

The prick of the needle will pain some more than others. While one man will say he scarcely feels it, another will cry out; but that should not disconcert the operator—the remedy must be inserted in the right place, or it will fail in accomplishing the desired result.

The acid will cause some inflammation and discomfort in some cases, but generally the patient can get up and go about his business.

It is best that the bowels be thoroughly moved and washed out with water, so that they will not move for about twenty-four hours. If the patient is nervous, and the operation gives pain, a dose of morphia might be given in order to allay pain and keep the bowels quiet the desired length of time.

But one tumor, especially if large, should be treated at once. If there are several tumors, one treatment every two weeks will soon cure them.

The above is called the "painless method" by traveling doctors, who are making large tees out of it in this and adjoining States.—*Med. and Surg. Rep.*

